# Integrated, Longitudinal Analysis of Cell-free DNA in Uveal Melanoma

**DOI:** 10.1158/2767-9764.CRC-22-0456

**Published:** 2023-02-15

**Authors:** Derek Wong, Ping Luo, Nadia Znassi, Diana P. Arteaga, Diana Gray, Arnavaz Danesh, Ming Han, Eric Y. Zhao, Stephanie Pedersen, Stephenie Prokopec, Yogi Sundaravadanam, Dax Torti, Kayla Marsh, Sareh Keshavarzi, Wei Xu, Hatem Krema, Anthony M. Joshua, Marcus O. Butler, Trevor J. Pugh

**Affiliations:** 1 Princess Margaret Cancer Center, University Health Network, Toronto, Ontario, Canada and Department of Immunology, University of Toronto, Toronto, Ontario, Canada.; 2 Department of Medicine, Division of Medical Oncology, University of Toronto, Toronto, Ontario, Canada.; 3 Ontario Institute for Cancer Research, Toronto, Ontario, Canada.; 4 Biostatistics Division, Dalla Lana School of Public Health, University of Toronto, Toronto, Canada.; 5 Department of Ocular Oncology, Princess Margaret Hospital, University of Toronto, Toronto, Canada.; 6 Department of Medical Oncology, Kinghorn Cancer Centre, St. Vincent's Hospital and Garvan Institute of Medical Research, Sydney, Australia.; 7 Faculty of Medicine, St. Vincent's Clinical School, University of New South Wales, Sydney, Australia.; 8 Department of Medical Biophysics, University of Toronto, Toronto, Ontario, Canada.

## Abstract

Uveal melanomas are rare tumors arising from melanocytes that reside in the eye. Despite surgical or radiation treatment, approximately 50% of patients with uveal melanoma will progress to metastatic disease, most often to the liver. Cell-free DNA (cfDNA) sequencing is a promising technology due to the minimally invasive sample collection and ability to infer multiple aspects of tumor response. We analyzed 46 serial cfDNA samples from 11 patients with uveal melanoma over a 1-year period following enucleation or brachytherapy (*n* = ∼4/patient) using targeted panel, shallow whole genome, and cell-free methylated DNA immunoprecipitation sequencing. We found detection of relapse was highly variable using independent analyses (*P* = 0.06–0.46), whereas a logistic regression model integrating all cfDNA profiles significantly improved relapse detection (*P* = 0.02), with greatest power derived from fragmentomic profiles. This work provides support for the use of integrated analyses to improve the sensitivity of circulating tumor DNA detection using multi-modal cfDNA sequencing.

**Significance:**

Here, we demonstrate integrated, longitudinal cfDNA sequencing using multi-omic approaches is more effective than unimodal analysis. This approach supports the use of frequent blood testing using comprehensive genomic, fragmentomic, and epigenomic techniques.

## Introduction

Uveal melanoma arising from melanocytes in the iris, ciliary body, or choroid (uveal tract) of the eye, is the most common intraocular tumor in adults and the second most common melanoma subtype after cutaneous melanoma (1). Despite surgical resection (enucleation) or radiation treatment (brachytherapy), approximately 50% of patients will progress to metastatic disease, most often to the liver. Once metastatic, there are currently few effective treatments and most patients survive fewer than 12 months (2). Recently, several clinical trials have shown improved survival outcomes in the metastatic setting such the phase II PEMDAC study of combined pembrolizumab and/or entinostat (3) and phase III trial of tebentafusp (4). Thus, early identification and stratification of patients at risk for metastasis is important for rapid enrollment and intervention, and ultimately, increased patient survival (5).

Genomically, uveal melanoma is characterized by few recurrently mutated genes. Over 80% of uveal melanomas harbor activating mutations of G-coupled paralog proteins GNA11 or GNAQ at codon 209, or less frequently, at codon 183 (6, 7). More recently, molecular profiling of uveal melanoma has led to two major prognostic subgroups defined by mutation and copy-number variants (CNVs; refs. 8–11). Patients most at risk of metastasis often harbor biallelic inactivation of *BAP1* and amplification of chromosome 8q. On the transcriptomic level, uveal melanoma progression and aggressiveness is driven by inactivation of both BAP1, a subunit of the polycomb-repressive deubiquitylase complex (8) and polycomb repressive complex 1 (PRC1) activity, leading to derepression of PRC1 target genes (12).

In recent years, circulating tumor DNA (ctDNA) has become an attractive biomarker due to noninvasive collection and sensitivity for identifying and assessing tumor-associated genomic aberrations. Combination of targeted ultra-deep sequencing (TS; ∼20,000X), and sequencing error suppression have been able to quantify mutant allelic fractions as low as 0.1% across a variety of cancer types (13–15). Tumor-associated chromosomal aberrations can also be reliably detected at a lower limit of 3% of the circulating DNA using shallow whole-genome sequencing (sWGS; ∼0.1–1X; ref. 16). However, both approaches rely on the presence of readily detectable genetic aberrations. More recently, studies using plasma sWGS have shown that cell-free DNA (cfDNA) fragmentation analysis can provide complementary information agnostic of genetic alterations. Cell-free fragmentomics is based on the observations that the release of cfDNA into the blood is a non-random process that is dependent on the chromatin architecture and epigenetic state of the cell of origin (17). Thus, analysis of cfDNA fragmentation patterns can be used to infer the cell types, such as cancer cells, that are contributing to the cfDNA milieu.

Another field of advancement in ctDNA detection is cell-free methylated DNA immunoprecipitation sequencing (cfMeDIP-seq) which enriches for 5-methylcytosine (5-mc; ref. 18). Methylation of DNA at CpG sites is an essential component of cell identity in both normal and cancer cells (19). These methylation marks are often conserved when released into the circulation and can be profiled to identify tissue- or cancer-specific methylation signatures in the plasma (20, 21). Because the defined biological mechanisms, high rate of hepatic metastasis and the low mutation burden of uveal melanoma, we hypothesize that uveal melanoma may be a good candidate for monitoring relapse using an integrated multi-modal ctDNA analysis approach.

In this study, we present a cohort of 11 newly diagnosed patients with uveal melanoma with up to five serial blood samples collected at baseline (pretreatment), and at 2 weeks, 3 months, 6 months, and 12 months after treatment with enucleation or brachytherapy. Using TS, sWGS, and cfMeDIP-seq, we profiled the ctDNA landscape and developed a customized multi-modal approach using genome, fragmentome, and methylome analyses to investigate differences in ctDNA trajectory in patients that relapse versus those that did not relapse.

## Materials and Methods

### Study Design and Patient Cohort

This study was conducted in accordance with recognized ethical guidelines (e.g., Declaration of Helsinki, CIOMS, Belmont Report, U.S. Common Rule). A total of 11 patients with newly diagnosed uveal melanoma were recruited at the Princess Margaret Cancer Center. All patients were enrolled with written informed consent and approved by the Institutional Review Board (REB# 15-9677). Tumor tissue was collected via fine needle aspiration and submitted to IMPACT genetics (https://impactgenetics.com/testing-services/uveal-melanoma-um/) for clinical copy-number analysis using multiplex ligation-dependent probe amplification (MLPA) on chromosomes 1p, 3, 6, and 8 (https://www.mrcholland.com/product/P027/676). Blood samples were collected into 3–4 10 mL tubes from each patient pretreatment (baseline) and 2 weeks, 3 months, 6 months, and 12 months following treatment. Buffy coats [peripheral blood mononuclear cells (PBMC)] were isolated from whole blood and used to validate somatic second hit mutations. All patients underwent routine clinical care by board-certified clinicians as per the standard of care. All samples were collected with informed consent for research and were approved by an Institutional Review Board.

### Blood Processing

Venous blood samples were collected in Ethylenediaminetetraacetic acid (EDTA) or Streck tubes (Streck). EDTA collection was processed within 2 hours. Whole blood samples were centrifuged at 4°C (1,900 × *g*, 10 minutes). PBMCs were separated from plasma and stored at −80°C. Isolated plasma was centrifuged a second time at 4°C (16,000 × *g*, 10 minutes) to remove residual cells and debris. Purified plasma was stored at −80°C until cfDNA extraction.

### DNA Extraction

DNA from blood was extracted using the QIAGEN QIAamp Circulating Nucleic Acid Kit (Qiagen). Genomic DNA from PBMCs was extracted using the DNeasy Blood and Tissue Kit (Qiagen). After extraction, genomic DNA was sheared to resemble cfDNA using an ultrasonicator (LE220, Covaris). Library preparation for TS, sWGS, and cfMeDIP was performed only for samples with >40 ng DNA yield. TS was excluded for samples with <40 ng DNA yield. Samples with <10 ng DNA yield were not processed.

### DNA Library Construction and Sequencing

Precapture libraries were prepared using KAPA Hyper Prep Kit (Kapa Biosystems) and xGen Duplex Seq Adapter-Tech Access (Integrated DNA Technologies). Unique molecular identifier (UMI)-ligated cfDNA libraries were then split for each assay (TS, sWGS, cfMeDIP-seq). To generate TS libraries, hybrid capture using a custom 38 kb panel of 318 probes targeting *BAP1, GNAQ, GNA11, SF3B1,* and *EIF1AX* (Supplementary Table S1) was performed on dual-indexed precapture libraries, then PCR amplified. sWGS and TS libraries were indexed, pooled, and sequenced on the NextSeq 500 using 150-bp paired-end sequencing reads (2 × 150 bp; Illumina). sWGS libraries were sequenced to a target 1X, and TS libraries were sequenced to a target 20,000X. cfMeDIP libraries were sequenced to a target 60 million clusters on the MiSeq Nano using 150-bp paired-end sequencing reads (2 × 150 bp; Illumina). UMI extraction and sequencing alignment to human genome reference GRCh38 was performed using Burrows-Wheelers Aligner version 0.7.12 (RRID:SCR_010910, https://github.com/oicr-gsi/bwa; ref. 22) and deduplicated using Samtools version 1.9 (RRID:SCR_002105) according to the following workflow: https://github.com/oicr-gsi/bwa. All sequencing coverage can be found in Supplementary Table S2. One buffy coat failed TS library construction and sequencing (Patient 06) due to low library diversity and was not used in downstream variant calling analysis.

### Cell-free Methylated DNA Immunoprecipitation

cfMeDIP for each sample was prepared as described previously (18) using 10 ng of UMI-ligated cfDNA library with the following deviations from the protocol. Briefly, 1 ng of *A. thaliana* DNA methylation control package containing one methylated and one unmethylated spike-in control BAC (Diagenode) was spiked into 10 ng of cfDNA library. A total of 5% of the sample was aliquoted as a control library. The remainder underwent immunoprecipitation using monoclonal antibody targeting 5-mc (Diagenode, clone #33D3, RRID:AB_2572207). Immunoprecipitated libraries and control libraries were amplified, indexed, and pooled. Cell-free methylated DNA immunoprecipitation and high-throughput sequencing (cfMeDIP-seq) data were analyzed using an integrated pipeline (MedRemix) which we will describe here (reproducible Snakemake pipeline code is available at https://github.com/pughlab/cfMeDIP-seq-analysis-pipeline). To extract UMI barcodes, the extract_barcodes.py from ConsensusCruncher package was executed on all fastq files (github linked below, May 19, 2021 commit; ref. 23). Reads were then aligned to the human genome (genome assembly GRCh38/hg38) using BWA-MEM v0.7.17 (RRID:SCR_010910) and sorted and indexed by Samtools v1.14 (RRID:SCR_002105; refs. 22, 24). Next, the depth of aligned fragments generated from paired reads of cfMeDIP-seq libraries was counted within nonoverlapping 300 bp windows, along with the number of CpGs within each window. The coverage depth was modeled as a two-component mixture, with the two components representing methylated and nonmethylated bins, accounting for CpG density and GC content by negative binomial regression. The mean coverage of nonmethylated reads was inferred from bins with zero CpGs as a function of GC content and treated as a fixed mixture component. The probability of methylation is a function of CpG density and coverage and thus represents two population of reads (methylated and unmethylated). To infer the mixing coefficients and the regression coefficients between the mean coverage of methylated bins and CpG density we used an expectation-maximization algorithm and iterated until convergence. In this process, each bin was assigned a posterior probability of being methylated.

### Targeted Sequencing Analysis

Aligned reads were error corrected and amalgamated using ConsensusCruncher (https://github.com/pughlab/ConsensusCruncher; ref. 23) according to the following workflow: https://github.com/oicr-gsi/consensusCruncherWorkflow. Germline variant calling was performed using The Genome Analysis Toolkit version 3.8 HaplotypeCaller (Broad Institute, RRID:SCR_001876; ref. 25). Somatic variant calling was performed using MuTect2 version 3.8 (Broad Institute, RRID:SCR_007073; ref. 26). Variant calling was performed individually on single-strand consensus sequences, duplex consensus sequences, and all_unique ConsensusCruncher outputs then merged. Variant allele frequency was annotated using read support from the all_unique ConsensusCruncher output. SNPs considered benign were removed from further analysis. Candidate pathogenic variants were manually reviewed using Integrative Genomics Viewer version 2.8.13 (RRID:SCR_011793, Illumina). Germline variants were classified according to the 2015 American College of Medical Genetics and Genomics guidelines (27).

### Copy-number Variant Analysis

GC correction, read count normalization, copy-number prediction, and tumor burden prediction were performed using the ichorCNA tool version 0.2.0 (Broad Institute; https://github.com/broadinstitute/ichorCNA; ref. 16) using the recommended default settings. To enrich for ctDNA, ichorCNA was performed once on all fragments and again using only short fragments (90–150 bp). This was performed because of increased detection of ctDNA reported in previous studies (28). The higher predicted tumor fraction (all vs. short) was used as the ichorCNA output. For short fragment analyses (90–150 bp), only samples with sWGS coverage > 0.1X after fragment pruning were used. For all ichorCNA analyses, only predicted tumor fractions > 0.03 are considered positive (based upon the ichorCNA limit of detection). Genome-wide z-scores were calculated binwise for each bin in chromosoems 3, 6, and 8 compared with healthy controls. A summed z-score was then generated for each sample weighted appropriately for the amount of bins in each chromosomal arm.

### Fragmentation Profile Analysis

Global fragment size distributions were calculated using Picard (RRID:SCR_006525) CollectInsertSizeMetrics (v4.0.1.2). Fragment size z-scores were calculated compared with healthy controls at each fragment length and then summed binwise for a genome-wide fragment size z-score. Genome-wide fragmentation profiles were generated using an adapted version of DELFI (https://github.com/pughlab/fragmentomics; ref. 29). Briefly, filtered and blacklist regions were compiled using GRCh38 coordinates. The ratio of short (90–150 bp) over long (151–220 bp) DNA fragments were calculated in 100 kb bins across the genome. GC content correction and read depth were corrected using loess with span ¾ separately for short and long fragments. GC-corrected read counts were compiled into 5 Mb bins, and the ratio of short/long reads were calculated and scaled to mean 0 with unit SD. PRC1 target loci were obtained from Bakhoum and colleagues (19) and used for PRC1 analysis. z-scores for fragmentation ratio profiles were calculated using binwise z-scores compared with healthy controls. z-scores were then summed for a genome-wide z-score.

Fragment coverage analyses were performed using the Griffin algorithm (https://github.com/adoebley/Griffin) as described in Doebley and colleagues (30). Liver and embryonic eye DNase hypersensitivity sites were obtained from the ENCODE project (https://www.encodeproject.org, RRID:SCR_006793; ref. 31). z-scores were calculated using the central coverage (± 30 bp) compared with healthy controls.

### Cell-free Methylome Analysis

Several metrics were calculated to control the quality of the samples. First, we counted the number of deduplicate read pairs using Picard (version 2.10.9), and all of our samples have more than 10 million deduplicated reads (median = 37.13, SD = 15.07). Second, the number of methylated and unmethylated Arabidopsis spike-ins aligned to F19K16 and F24B22 were counted, respectively, and the fraction of methylated spike-ins out of the total spike-ins was used to assess enrichment efficiency. No sample was removed because they have reasonable fraction of spike-in reads that were methylated (93.11%–99.35%). Finally, GoGe and relH enrichment scores calculated by MEDIPS (1.42.0) were used to access CpG enrichment efficiency. The minimum GoGe and relH scores are 1.80 and 2.83, respectively, and we retained all the samples for subsequent analysis.

To identify uveal melanoma–specific hypermethylated CpG sites, The Cancer Genome Atlas (TCGA; RRID:SCR_003193) DNA methylation data measured by Illumina HumanMethylation450k array were downloaded from the GDC legacy archive (RRID:SCR_014514). We only downloaded cohorts with more than 50 samples, resulting in 33 datasets in total. Pairwise differential methylation analysis was performed by Limma (RRID:SCR_010943) for uveal melanoma and the other 32 cancer cohorts as well as a normal blood cohort (downloaded from GSE102468) using 50 randomly selected samples from each dataset (32). Hypermethylated cytosines with higher methylation in uveal melanoma versus an individual comparison were defined by a log fold change ≥2 and a *P* value adjusted for multiple comparisons (Benjamini–Hochberg) <0.05. A hypermethylated cytosine was considered as uveal melanoma–specific only if it was hypermethylated in more than 21 pairs of comparisons. Finally, 77 CpG sites were identified as uveal melanoma–specific signatures.

The uveal melanoma–specific CpG sites were mapped to the methylation bins based on their chromosomal positions, resulting in 77 bins. The methylation score of a sample is defined as the cumulation posterior probabilities of the 77 bins obtained by MedRemix. The higher the score, the more possible that the 77 CpG sites are methylated in the corresponding sample, which means the patient is more likely to be cancer positive.

### Healthy Control Cohorts

Healthy blood controls were generated from both in house and previously published studies (28, 29, 33, 34). Written informed consent was obtained for all in-house healthy controls and was recruited with Institutional Review Board approval (REB#: 19-6239). In-house healthy controls underwent TS (*n* = 11), sWGS (*n* = 11), and cfMeDIP-seq (*n* = 14). All healthy control data were aligned to GRCh38 as described above and processed according to GATK (RRID:SCR_001876) best practices. Healthy control plasma analyses were performed the same as described above.

### Integration Analysis

Integration of z-score values for copy number, insert size, genome-wide fragment ratio, PRC1 fragment ratio, fragment coverage across liver open chromatin sites, and uveal melanoma methylation was performed using a mixed-effect logistic regression model. The model was trained excluding patients that were lost to follow-up using 10-fold cross-validation. Baseline and timepoints at- or post-relapse were classified as “ctDNA positive” while all other timepoints were classified as “ctDNA negative” for training classification purposes. Final predictions were generated for all samples including patients lost to follow-up using the trained model. Individual analysis performances were calculated using a univariate mixed effect logistic regression model. Model generation, classification, and regression analyses were all performed using the caret package in R (v4.0.3, RRID:SCR_021138; ref. 35).

### Data and Code Availability

All sequencing data files (TS, sWGS, cfMeDIP) are deposited in the European Genome-Phenome Archive (EGA) under the accession number EGAD00001008998. Code and processed files to reproduce all analyses and figures are available at https://github.com/pughlab/TGL48_Uveal_Melanoma

## Results

### Patient Cohort

Patients with newly diagnosed uveal melanoma were recruited from the Princess Margaret Cancer Center, 7 males and 4 females. Tumor tissue was collected using fine needle aspiration prior to receiving brachytherapy (7/11) or enucleation (4/11) as standard of care and submitted for clinical copy-number analysis using MLPA. Patients were followed between the period of January 17, 2017 and April 01, 2022. During this period, 6 patients relapsed (5 brachytherapy and 1 enucleation; median disease-free survival = 9.9 months). Of these 6 patients, 4 relapsed within the 12-month period of plasma collection (2 patients relapsed at 24.9 and 60.0 months) and 4 succumbed to their disease (median overall survival = 20.4 months). Patient 01 relapsed 60 months following treatment and thus for the genomic analyses, this case was included in a group of nonrelapse/late-relapse patients. Table 1 summarizes clinical patient information and clinical copy-number profiles.

**TABLE 1:** Clinical information for our patient cohort

ID	Sex	Age at diagnosis	Tumor size (mm)	Side	Stage	Treatment	Lost to follow-up	Relapse	Relapse site	Relapse (months)	Death (months)	Follow-up (months)	Other diagnoses (months)	1p	3	6p	6q	8p	8q
UMB-009	F	78.3	5.9 × 15.0 × 15.0	R	IIB T2b	Brachytherapy	—	Yes	Liver	3.1	16.3	16.3	Bipolar, Dyslipidemia, Hypothyroidism, Parkinson	—	LOH	ROH	LOH	LOH	Gain
UMB-003	M	62.5	10.0 × 16.4 × 18.0	R	IIB T3a	Brachytherapy	—	Yes	Liver	6.0	11.5	11.5	Papillary carcinoma of the thyroid (4.0)	—	LOH	—	—	LOH	Gain
UMB-005	M	71.9	10.8 × 19.4 × 17.0	L	IIIA T3b	Brachytherapy	—	Yes	Liver	7.5	23.4	23.4	Coronary heart disease	—	LOH	—	—	—	Gain
UMB-004	M	53.5	15.0 × 21.0 × 21.0	L	IIIB T4b	Enucleation	—	Yes	Liver	12.3	37.7	37.7	—	LOH	LOH	—	LOH	LOH	Gain
UMB-006	F	22.3	7.3 × 14.8 × 16.6	L	IIIA T3b	Brachytherapy	—	Yes	Liver	24.9	58.9	58.9	Asthma	—	—	—	—	—	—
UMB-001	M	65.7	7.8 × 19.0 × 19.3	R	IIIB T4b	Brachytherapy	—	Yes	Liver	60.0	—	62.9	Grade 1 follicular lymphoma (4.0)	—	LOH	—	—	—	—
UMB-002	F	78.1	6.4 × 13.1 × 11.7	L	IIB T3a	Brachytherapy	—	No	—	—	—	48.2	Hypertension	—	—	—	—	—	—
UMB-011	M	57.5	7.7 × 16.4 × 16.4	L	IIB T3a	Brachytherapy	—	No	—	—	—	54.5	Squamous cell carcinoma of the neck (24.0)	—	—	ROH	—	—	—
UMB-007	M	59.4	15.0 × 16.0 × 16.0	R	IIIB T4b	Enucleation	Yes	—	—	—	—	6.3	—	—	LOH	—	LOH	LOH	Gain
UMB-008	F	54.5	12.0 × 8.0 × 8.0	L	IIIA T3c	Enucleation	Yes	—	—	—	—	47.1	HIV, Hepatitis C, Asthma/COPD, Hypertension	LOH	LOH	Gain	—	Gain	Gain
UMB-010	M	76.7	16.0 × 16.0 × 16.0	R	IIIA T4a	Enucleation	Yes	—	—	—	—	12.2	Hypertension, Fibromyalgia	—	—	—	—	—	Gain

**FIGURE 1:**
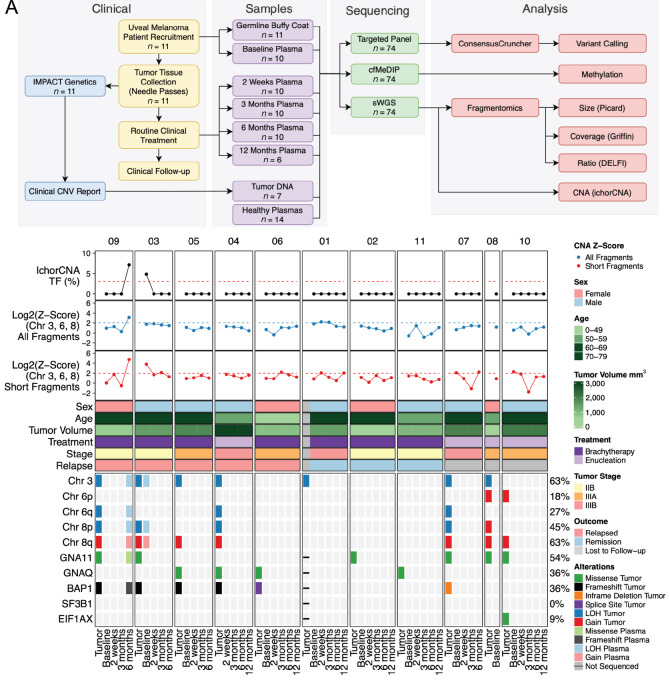
Mutations and copy-number alterations. **A,** A flowchart schematic outlining the workflow for this study. **B,** Oncoplot showing mutations and copy-number alterations identified in patient tumors and plasmas. IchorCNA tumor fraction represents the predicted tumor fraction of all or short fragment analysis, whichever is higher. Copy-number z-scores were calculated using copy-number ratio outputs from ichorCNA using only chromosomes 3, 6, and 8. Dashed red line represents the threshold of healthy and cancer based upon the 90th percentile of the healthy controls. Relapsed patients are ordered by time to relapse (shortest to longest) with arrows denoting the timepoint closest to relapse.

Baseline plasma was not collected for 1 patient (Patient 04), and 1 patient was discontinued following baseline plasma collection due to untreated human immunodeficiency virus (HIV; Patient 08). Patient 07 was also lost to follow-up at 6 months (12-month plasma not collected) and similarly Patient 10 was lost to follow-up following their 12-month plasma collection. In addition, plasma timepoints were not collected for Patients 09, 03, and 05 following relapse (12-month plasma not collected). In total, 46 plasma samples were collected from our 11 patients. Sample schedules for each patient are listed in Supplementary Table S3. A flowchart of our study design is displayed in Fig. 1A.

### Detection of Uveal Melanoma Mutations and Copy-number Alterations in ctDNA

To determine whether somatic mutations and copy-number variants (CNVs) are detectable in the cfDNA from patients with uveal melanoma at diagnosis and prior to relapse, we performed TS and sWGS on matched plasma cfDNA, germline buffy coat genomic DNA, and seven available tumor tissues at diagnosis (four tumors exhausted by MLPA assay). Mutation data were available for 10 of 11 tumors using TS data (7/11) and reflexive clinical testing (5/11). In the baseline tumors, we identified mutations in *GNA11* (6/10), *GNAQ* (4/10), *BAP1* (5/10), and *EIF1AX* (1/10). Chromosome 3 monosomy (7/11), 8p monosomy (4/11), 6p gain (2/11), and 8q gain (7/11) were identified in the tumor tissues by IMPACT genetics (Fig. 1; Supplementary Table S4). In patients with disease relapse (*n* = 5), all (5/5) harbored mutations in *BAP1* and near universal concurrent chromosome 3 monosomy and 8q amplification (4/5). Conversely, patients that did not relapse (*n* = 3) did not harbor *BAP1* alterations or 8q amplification (Patient 01 had chromosome 3 monosomy).

In contrast to the tissue samples, TS only identified mutations in *GNA11* [variant allele frequency (vaf) = 0.92%] and *BAP1* (vaf = 1.57%) at one plasma timepoint in 1 patient with metastatic disease 6 months after brachytherapy (Patient 09—6 months). Mutations were not identified in any baseline plasma samples (Fig. 1B).

Comparing clinical copy-number findings from tumor samples using MLPA with sWGS copy-number analysis using ichorCNA, we found near perfect concordance (McNemar *χ*^2^ = 0, df = 1, *P* = 1). In Patient 08, MLPA found a borderline chromosome 3 loss (allelic imbalance ratio = 1.82) while ichorCNA predicted the region to be copy neutral (tumor fraction = 0.62, logR copy number = −0.098; Supplementary Fig. S1A). This discrepancy may be due to the specific MLPA probes used versus whole chromosome analysis by ichorCNA.

In plasma cfDNA samples, we identified 0 cancer positive samples (tumor fraction > 0.03) using the standard ichorCNA workflow. However, after rerunning ichorCNA using only short fragments (<150 bp), an approach which has been shown to enrich for ctDNA (28, 36), we detected two cancer positive samples (Patient 03—baseline tumor fraction = 0.052 and Patient 09—6 months tumor fraction = 0.074; Supplementary Fig. S1B and S1C). These two samples were also the only samples where CNVs predicted by ichorCNA were concordant with CNVs identified in their respective tumors (Supplementary Fig. S1D). To explore additional metrics of detecting CNVs, we calculated z-scores using the ichorCNA copy-number ratio outputs for chromosomes 3, 6, and 8 which are recurrently altered in uveal melanoma. Our z-score approach showed significant elevation of Patient 03—baseline (all fragment z-score = 3.38, short fragment z-score = 13.69) and Patient 09—6 months (all fragment z-score = 8.95, short fragment z-score = 26.49) compared with healthy controls (mean all fragment z-score = 2.40, SD = 1.21 and mean short fragment z-score = 2.40, SD = 1.11; Fig. 1B). No other samples showed significantly elevated z-scores.

In Patient 10, both short and full fragment ichorCNA analyses predicted tumor fractions of approximately 0.10 in plasma and lymphocyte samples taken at all timepoints. In these samples, the tumor fraction prediction was based solely on a gain of chromosome 15, an event that has been associated with clonal hematopoiesis of indeterminate potential (CHIP; ref. 37). The low enrichment for tumor-associated CNVs combined with the lack of mutations detected in the plasma suggest that the limits of detection for targeted panel and ichorCNA analyses may not be sensitive enough in our cohort. In addition, these genomic analyses, especially CNV detection using ichorCNA can be obscured by age-related genetic alterations such as CHIP in tumor uninformed analyses.

### Patients that Relapse Exhibit Cancer-associated Fragmentation Profiles

**FIGURE 2:**
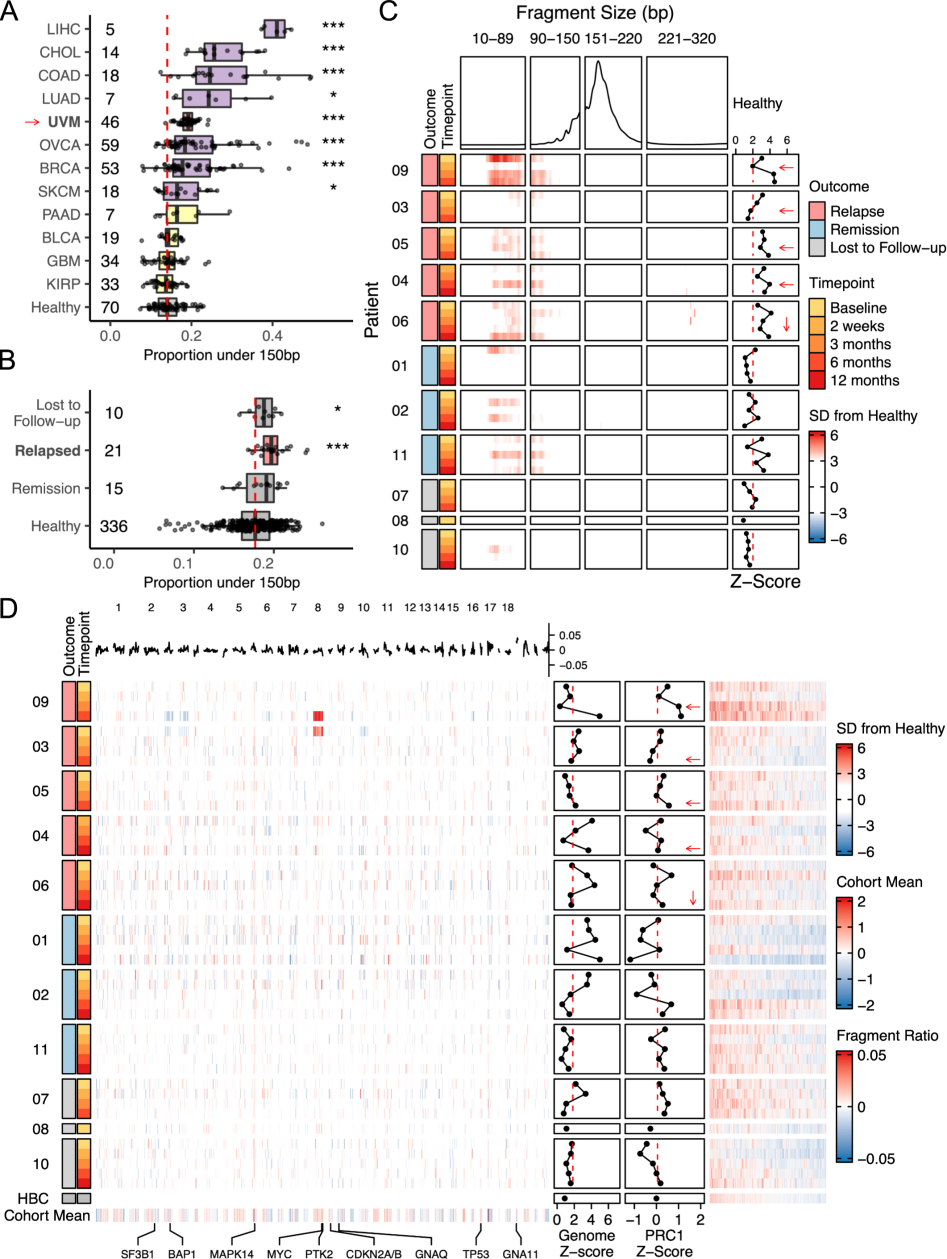
Cell-free fragmentation profiles. **A,** Boxplots comparing the proportion of short DNA fragments (10–150 bp) over all DNA fragments (10–250 bp) in uveal melanoma compared with several cancer types and healthy controls from the Mouliere and colleagues study (14). **B,** Boxplots comparing the proportion of short DNA fragments (10–150 bp) over all fragments (10–250 bp) based on clinical outcome (relapse vs. remission) compared with a cohort of in-house and external healthy controls. **C,** A heatmap showing the distance from the healthy median for each fragment length across the cfDNA fragment size distribution. The distribution of the healthy median is displayed above. z-scores were calculated using fragments between 90 and 150 bp. **D,** Left: A heatmap of genome-wide fragmentation profiles showing the distance from the healthy median. The healthy median profile is displayed on the left, and the cohort mean distance from the healthy is shown on the right. z-scores were calculated versus a cohort of healthy controls. Right: A heatmap of fragmentation ratios overlapping with PRC1 target loci. z-scores were calculated versus a cohort of healthy controls. *, *P* > 0.05; **, *P* > 0.01; ***, *P* > 0.001. Dashed red line represents the threshold of healthy and cancer based upon the 90th quantile of the healthy controls. Relapsed patients are ordered by time to relapse (shortest to longest) with arrows denoting the timepoint closest to relapse.

To further explore other modes of detecting cancer-associated signals, agnostic of genomic alterations, in our cohort, we investigated the cell-free fragmentome measured by sWGS. On a global level, a higher proportion of short (<150 bp) DNA fragments was observed in our uveal melanoma cohort (median = 0.19, *n* = 46) compared with healthy controls (median = 0.14, *n* = 70) and a compendium of tumor types (Fig. 2A; ref. 36). Patients that relapsed exhibited an even greater proportion of short fragments and greater deviation from the healthy distribution compared with patients that did not relapse (*P* = 0.035; Fig. 2B; Supplementary Fig. S2A).

To visualize the change in DNA fragment length distributions over time, we calculated z-scores using the frequency of DNA fragments between 90 and 150 bp compared with a cohort of healthy controls (Fig. 2C). In 4 of 5 patients that relapsed we observed consistently elevated z-scores (more short fragments) across all timepoints and increases in z-score prior to or at relapse. In 2 patients (Patient 04 and 06), an increase in z-score was detected 6 and 12 months prior to relapse, respectively. In contrast, nonrelapsed patients did not show significant changes during follow-up: Patient 01’s z-scores decreased to within the range of healthy-controls following treatment while Patients 02 and 11 fluctuated between elevated and healthy with no clear pattern. While patients with uveal melanoma were observed to exhibit an increase in short fragments using this global approach, these changes are subtle and not as pronounced compared with other cancer types (uveal melanoma relapse: median = 0.20, SD = 0.02 vs. Lung Adenocarcinoma (LUAD): median = 0.24, SD = 0.09, Colorectal Adenocarcinoma (COAD): median = 0.25, SD = 0.11, Liver Hepatocellular Carcinoma (LIHC): median = 0.41, SD = 0.03; ref. 36).

Next, to investigate the influence of the uveal melanoma chromatin architecture on the release of cfDNA fragments, we generated fragmentation profiles by calculating the ratio of short (90–150 bp) to long (151–220 bp) fragments binned across the genome (Supplementary Fig. S2B; ref. 29). z-scores were calculated at each genomic bin compared with a cohort of healthy controls and a genome-wide z-score was calculated using a summation of binwise z-scores (Fig. 2D; Supplementary Fig. S2C). In 3 of 5 patients that relapsed (Patients 09, 05, and 04), we observed increasing genome-wide fragmentation z-scores through post-treatment timepoints. Conversely, in 2 of 3 patients that did not relapse, fragmentation profiles resembled those of healthy controls following treatment. In 2 patients, Patient 03 at baseline and Patient 09 at 6 months posttreatment, decreased fragment ratios were detected in chromosome 3 (fewer short fragments) and increased fragment ratios were detected in chromosome 8q (more short fragments). These changes were wholly concordant with copy-number alterations detected in the matched tumors. However, fragment ratios along chromosome 15 did not differ between Patient 10, where we identified a CHIP-associated gain, compared with the remainder of the cohort (Supplementary Fig. S2D). This provides further support that fragment ratios are useful for detecting tumor-specific signal and are not obfuscated by CHIP-associated findings (38, 39). Investigating cohort-wide changes in fragment ratio, we observed a focal increase of the mean fragment ratio at a locus of chromosome 8q across the cohort. This locus harbors the oncogenes *MYC,* a commonly amplified uveal melanoma gene (40), and *PTK2*, a regulator of metastasis in uveal melanoma (41). Further studies using larger cohorts or metastatic patients may reveal additional genome-wide fragmentomic signatures associated with early uveal melanoma relapse.

Changes in the chromatin landscape of tumor cells can often be triggered by a singular pathway or event such as inactivation of PRC1 activity in uveal melanoma (12). To determine whether we could detect changes in fragmentation at PRC1 binding sites, we investigated the fragment ratios at 1,895 100 kb genomic bins that overlapped with PRC1 binding sites (*n* = 5,340; ref. 12). Patients that relapsed had higher fragment ratios (increased short fragments, scaled median = 0.0049) compared with patients that did not relapse (scaled median = 0.00014; Supplementary Fig. S2E and S2F). Interestingly, increased fragment ratios at PRC1 target loci could be detected in Patient 09 and Patient 04, 3 and 6 months prior to genome-wide detection, respectively (Fig. 2D) which was not observed in randomly selected bins (Supplementary Fig. S2G). However, we also observed variability in both genome-wide and PRC1 fragment ratio scores in patients that did not relapse. Fragment ratio scores showed similar trends between genome wide and PRC1 targets with better detection performance by PRC1 target loci in 3 of 5 patients that relapsed (Patient 05, 06, and 09; Supplementary Fig. S2H). These data suggest that biologically informed fragmentomic analyses can enable inference of prognostically relevant biology that would not be captured by genomic and genome-wide analyses.

**FIGURE 3:**
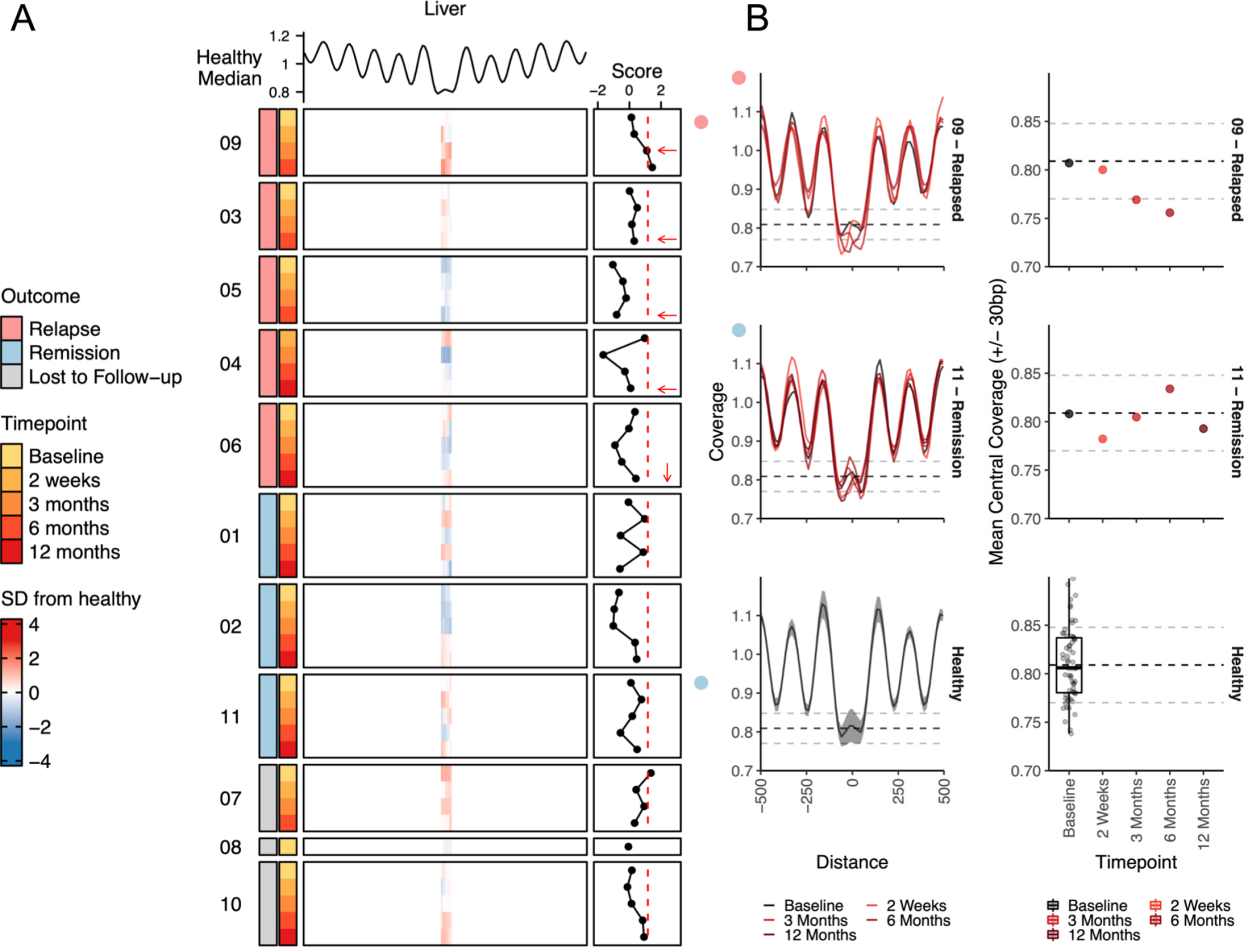
Cell-free nucleosome occupancy profiles. **A,** Heatmap showing the distance from the healthy median for nucleosome profiles calculated on liver-specific DNase hypersensitivity sites (±30 bp central coverage). The healthy median is displayed above. z-scores were calculated versus a cohort of healthy controls. Dashed red line represents the threshold of healthy and cancer based upon the 90th quantile of the healthy controls. Relapsed patients are ordered by time to relapse (shortest to longest) with arrows denoting the timepoint closest to relapse. **B,** Left: Nucleosome positioning curves of liver-associated open chromatin sites for patient 09 (relapsed), patient 11 (remission), and a cohort of healthy controls. Shaded area around the healthy control represents ± 1 SD. Right: Dot-plot showing the mean central coverage (±30 bp) of liver-associated open chromatin sites. Dashed line shows the mean coverage and ± 1 SDs of a cohort of healthy controls.

In addition to fragment ratio, the abundance and patterns of cfDNA coverage across the genome has also been shown to be effective for inferring cancer status (42), cancer type (30), and transcriptional activity (43, 44). This is due to the influence of nucleosome occupancy on the release of DNA fragments into the plasma (45). Coverage most often peaks at sites occupied by nucleosomes and drops at sites with open chromatin and linking DNA. Using the Griffin tool (30), we calculated coverage profiles for open chromatin sites associated with the liver (Fig. 3A) and embryonic eye (Supplementary Fig. S3A and 3B; refs. 31, 46). Liver was chosen because liver metastases are the most common relapse site for uveal melanoma and were found in all patients that relapsed in our cohort. Studies have shown that liver damage caused by lifestyle factors, illness, or cancer metastases can result in the destruction and release of normal liver DNA into the bloodstream (47–49). Patient 09 who relapsed to the liver at 5 months showed the most notable decrease in coverage at liver-associated open chromatin sites at follow-up timepoints suggestive of increased contribution of liver DNA to the cfDNA milieu (Fig. 3B). Analysis of clinical information also did not identify liver-associated diseases except uveal melanoma relapse that could contribute to the liver signal observed. This pattern of decreased coverage compared with healthy controls was not observed in open chromatin sites associated with other organs (Supplementary Fig. S3C). In patients that did not relapse and healthy controls, we did observe variable scores which may be due to changes or fluctuations in liver contribution due to lifestyle factors.

### Uveal Melanoma–associated Methylation is Enriched in Metastatic Patients

To investigate whether analysis of methylation signatures in the plasma can be used to detect the abundance of uveal melanoma ctDNA, we performed cfMeDIP-seq. Evaluating quality control (QC) metrics, all samples showed a high enrichment of methylated *Arabidopsis* spike-in controls (93.11%–99.35%) and CpG enrichment efficiency (relH = 2.83–4.01, GoGE = 1.80–2.18; Supplementary Fig. S4A). To evaluate for uveal melanoma–specific methylation, we first built an uveal melanoma–specific methylation signature (77 CpG sites; Supplementary Table S5) using methylation array data from The Cancer Genome Atlas (TCGA) uveal melanoma dataset (*n* = 50, encoded as UVM) compared to all TCGA cancer cohorts (*n* = 32), and a cohort of healthy blood controls (*n* = 50). We then applied this signature to methylated DNA immunoprecipitation (MeDIP) data from our uveal melanoma tumor samples (*n* = 7) as well as cell-free MeDIP (cfMeDIP-seq) data from 46 matched plasmas and 14 healthy plasma controls. As expected, all seven tumor samples scored highly for the uveal melanoma methylation signature (median = 65.21, SD = 2.55) while healthy control plasmas received low scores (median = 21.22, SD = 2.17; Supplementary Fig. S4B).

**FIGURE 4:**
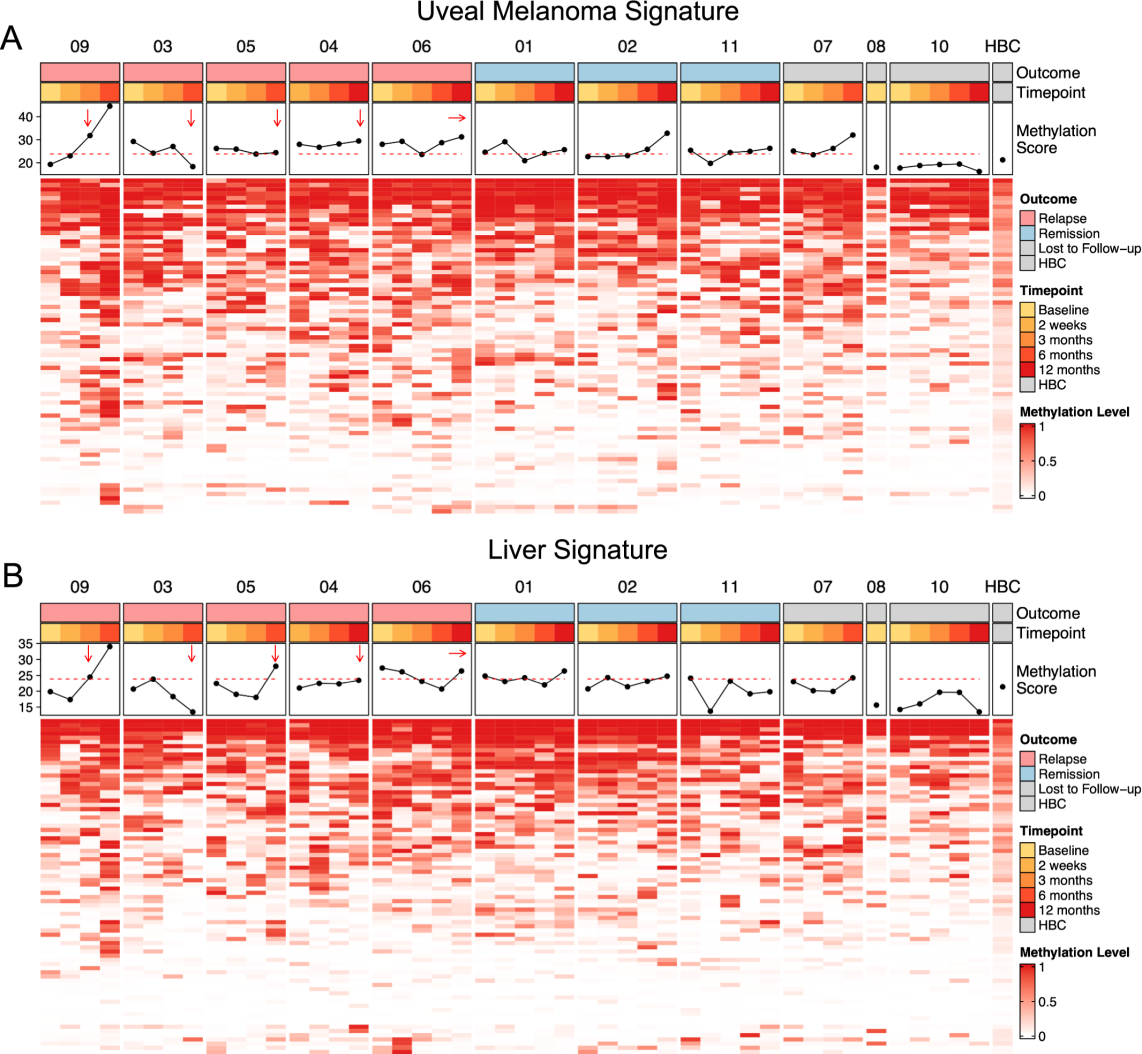
Cell-free methylation profiling. **A,** Heatmap showing enrichment for uveal melanoma–specific methylation signal. Methylation scores were calculated by a summation of enrichment at uveal melanoma–specific methylation sites. Dashed red line represents the 90th quantile of the healthy controls. **B,** Heatmap showing enrichment for healthy liver-specific methylation signal. Methylation scores were calculated by a summation of enrichment at liver-specific methylation sites. Dashed red line represents the 90th quantile of the healthy controls. Relapsed patients are ordered by time to relapse (shortest to longest) with arrows denoting the timepoint closest to relapse.

Only one relapsed patient (Patient 09) showed a significant increase in plasma methylation score at 6 months (score = 44.57; Fig. 4A). By this timepoint, the patient had already been diagnosed with metastatic disease suggesting that our uveal melanoma signature may not be sensitive enough to reliably detect nonmetastatic disease. In total, 3 of 5 patients who relapsed had methylation scores that increased following treatment. However, 2 of 3 nonrelapse patients also displayed this pattern. This lack of sensitivity and specificity may be a result of the limited number of CpGs captured in our uveal melanoma signature due to using methylation array data. Given a large enough dataset of tumor samples and genome-wide methylation information such as bisulphite sequencing or MeDIP, a more powerful uveal melanoma signature may be built.

We performed a similar analysis using a liver-specific methylation classifier to determine whether liver-derived DNA could be detected in patients that relapsed to the liver (all patients). Similarly, Patient 09 at 6 months scored the highest (score = 34.01), consistent with our fragmentation analyses (Fig. 4B). Patients 05 (score = 27.87) and 06 (score = 26.37) also showed increasing scores prior to relapse, though not significantly higher compared with nonrelapse patients (median = 23.11, SD = 3.10) or healthy controls (median = 23.11, SD = 3.10). These trends suggest that profiling of the cell-free methylome may be useful for detecting liver metastases; however, serial profiling and establishing a patient-specific baseline may be required because of the variable contribution of liver-derived cfDNA to the cfDNA milieu due to lifestyle and genetic factors (50, 51).

Finally, to determine whether methylation analyses may also be affected by CHIP, we assessed the methylation probabilities in all bins from chromosome 15 and found that the methylation values from Patient 10 plasma samples were not significantly different compared with all other patients (Supplementary Fig. S4C). This finding suggests that similar to fragmentomic analyses, cfMeDIP-seq analysis is unaffected by CHIP-associated CNVs.

### Integration of ctDNA Metrics Improves Robustness of ctDNA Detection

**FIGURE 5:**
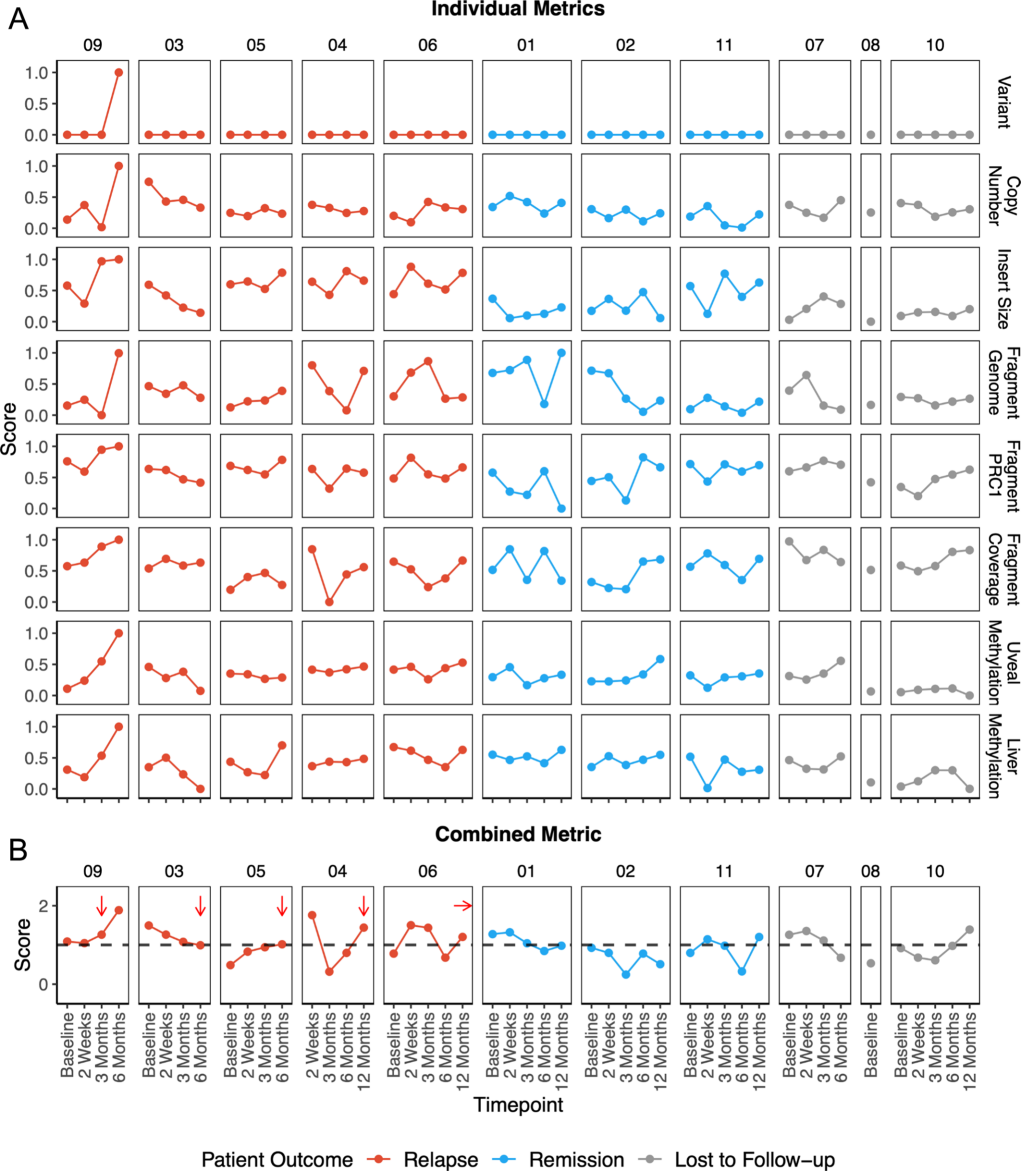
Integration of analysis metrics. **A,** z-score metrics for individual analyses scaled from 0 to 1. **B,** Integrated z-score metric calculated from all individual metrics from above, excluding variant analysis, using logistic regression. Scores were normalized to the mean cohort baseline represented by the dashed black line. Relapsed patients are ordered by time to relapse (shortest to longest) with arrows denoting the timepoint closest to relapse.

Individually, we demonstrated that each of our investigations are able to detect cancer-associated signals, but often lacked sensitivity (mutation and copy number), specificity (genome-wide fragment ratio), or suffered from excess noise (Fig. 5A). This variability may be due to the low ctDNA fractions in our samples. The only patient that showed a consistently robust ctDNA signal in every analysis (Patient 09) was also the only patient where a plasma sample was collected after metastasis. In the remaining patients, we observed inter-analysis variability suggesting that certain analyses performed better in different patients. To reduce the inter-analysis variability between our patients and of individual metrics, and to reveal ctDNA trends in our cohort, we explored integration of genome, fragmentome, and methylome analyses. Variant analysis was excluded from integration due to the limited number of cfDNA specimens with detectable mutations. Pairwise comparisons of individual analyses (copy number, fragment size, fragment ratio, PRC1 fragment ratio, uveal melanoma methylation, liver methylation) showed variable correlations (*R*^2^ = −0.26 to 0.75) which suggests that each analysis may provide independent biological readouts (Supplementary Fig. S5). Integration was performed by applying a mixed-effect logistic regression (Gaussian distribution) to the scores calculated for each individual analysis and then normalized to the mean cohort baseline (Fig. 5B). This normalization was chosen to account for differences in baseline ctDNA burden and a lack of baseline plasma for Patient 04. Univariate analysis of each metric using a mixed-effects logistic regression confirmed that the integrated score performed better (*P* = 0.02) compared with individual analyses (*P* range, 0.06–0.38; Supplementary Table S6).

In 3 of 4 patients that relapsed (Patient 09, 05, 04) within our collection timeframe (12 months), we observed a trend of increasing integrated scores preceding relapse. Globally, 4 of 5 patients that relapsed showed an increasing integrated ctDNA signal posttreatment. In 3 of 3 patients that did not relapse, we observed variable integrated scores that maintained below or close to the baseline with no clear pattern of ctDNA signal increase. Finally, for patients that were lost to follow-up and had serial samples, Patient 07 had decreasing integrated ctDNA signal over time, while Patient 10 exhibited increasing integrated ctDNA score over time post-treatment which may suggest that Patient 10 eventually relapsed.

## Discussion

Here, we present an integrated analysis of 46 cfDNAs profiled using genomic, fragmentomic, and epigenomic methods in a cohort of 11 patients with uveal melanoma with serial pre- and post-intervention timepoints. Each assay (TS, sWGS, cfMeDIP-seq) enables analyses that measure independent biological signals which when integrated, can improve the overall sensitivity, specificity, and robustness of predictions.

In this cohort, the lack of mutations detected by TS may reflect the low tumor burden in localized uveal melanoma tumors and thus low levels of ctDNA released into the bloodstream (52). While downstream bioinformatic analysis techniques have significantly increased the sensitivity of mutation detection by TS (23, 53), this strategy is fundamentally limited by the reliance on idealized detection of mutations at few loci and the number of genomic equivalents available in small quantities of cfDNA (33). In patients with metastatic uveal melanoma, TS and digital droplet PCR have been shown to be effective in predicting treatment response suggesting that sufficient ctDNA release occurs in the metastatic setting (54, 55). Similarly, copy-number alteration detection in the plasma, using ichorCNA, was restricted by the limit of detection required for confident CNV detection (∼3%–5% tumor fraction; refs. 16, 56).

Compared with TS and copy-number analysis, fragmentomic analysis of sWGS data was more effective at detecting ctDNA abundance in patients preceding relapse in our study and not affected by CHIP-associated findings. Incorporation of sWGS also allowed for analysis of many more features (copy number, fragment size, fragment ratio, fragment coverage) which greatly increased the breadth of metrics available for integration. This finding is consistent with other studies illustrating effective early cancer detection and classification in other cancer types using fragmentomic analyses (29, 30, 36, 43, 45).

Some caveats to this study are the small sample size (*n* = 11), limited timeframe, and findings of secondary cancer diagnoses during clinical follow-up. Of the patients that relapsed, only four relapsed within our plasma collection timeframe. Two patients relapsed at 24 and 60 months posttreatment suggesting that extended monitoring may have been beneficial for these “late relapsers.” During clinical follow-up of our patients, secondary cancer diagnoses included grade 1 follicular lymphoma (4 months after uveal melanoma diagnosis), local recurrence of a prior papillary thyroid carcinoma (4 months after uveal melanoma diagnosis), and stage 1 squamous cell carcinoma (2 years after uveal melanoma diagnosis) in Patients 01, 03, and 11, respectively. These secondary neoplasms may have contributed to the elevated ctDNA signal we observed in Patients 01 and 11, especially for global, nonspecific analyses such as fragment size and genome-wide fragment ratio.

While many ctDNA studies are currently focused on singular or bimodal approaches to ctDNA detection, integrated multi-modal analyses should be explored further to assess the relative benefits of including additional assays, analyses, and biological information. While our cohort is small, this study demonstrates that integration of multiple plasma-based analyses may provide a more holistic view into the ctDNA landscape in low tumor burden settings such as uveal melanoma compared with single-modal analyses. Further studies with larger cohorts will be necessary for creating more robust machine learning models and classifiers to validate this integration approach.

## Supplementary Material

Table TS1 panel design

Table TS2 sequencing coverage

Table TS3 sample schedule

Table TS4 mutations

Table TS5 differentially methylated probes

Table TS6 regression statistics

Figure S1 Supplemental Figure 1

Figure S2 Supplemental Figure 2

Figure S3 Supplemental Figure 3

Figure S4 Supplemental Figure 4

Figure S5 Supplemental Figure 5
