# Supplementary material for: Integrated, Longitudinal Analysis of Cell-free DNA in Uveal Melanoma
Source: Cancer Res Commun. 2023 Feb 15;3(2):267–80. doi: 10.1158/2767-9764.CRC-22-0456 (PMC9973415; doi:10.1158/2767-9764.CRC-22-0456)
Supplement: Figure S1 — Supplemental Figure 1 [file crc-22-0456-s07.pdf]

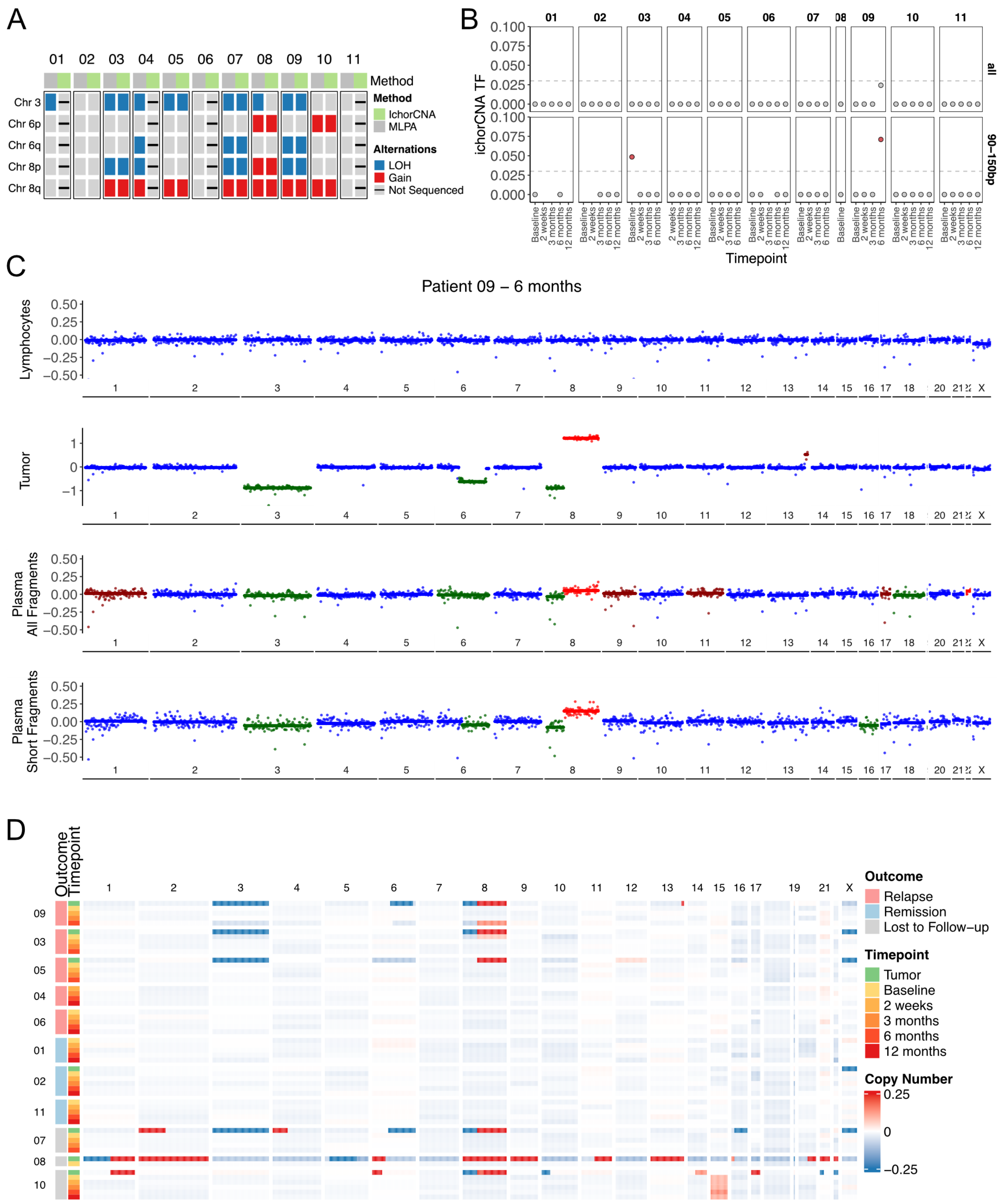

Supplemental Figure 1:

A) Tumor copy number comparison between MLPA and ichorCNA.

B) Comparison of ichorCNA values using all fragments and short fragments (90-150bp).

C) IchorCNA tracks for Patient 09 – 6 months showing enrichment of copy number alterations through short fragment analysis compared to tumor and lymphocytes.

D) IchorCNA log2copy number ratios for tumor and plasma samples.
