# Supplementary material for: Integrated, Longitudinal Analysis of Cell-free DNA in Uveal Melanoma
Source: Cancer Res Commun. 2023 Feb 15;3(2):267–80. doi: 10.1158/2767-9764.CRC-22-0456 (PMC9973415; doi:10.1158/2767-9764.CRC-22-0456)
Supplement: Figure S2 — Supplemental Figure 2 [file crc-22-0456-s08.pdf]

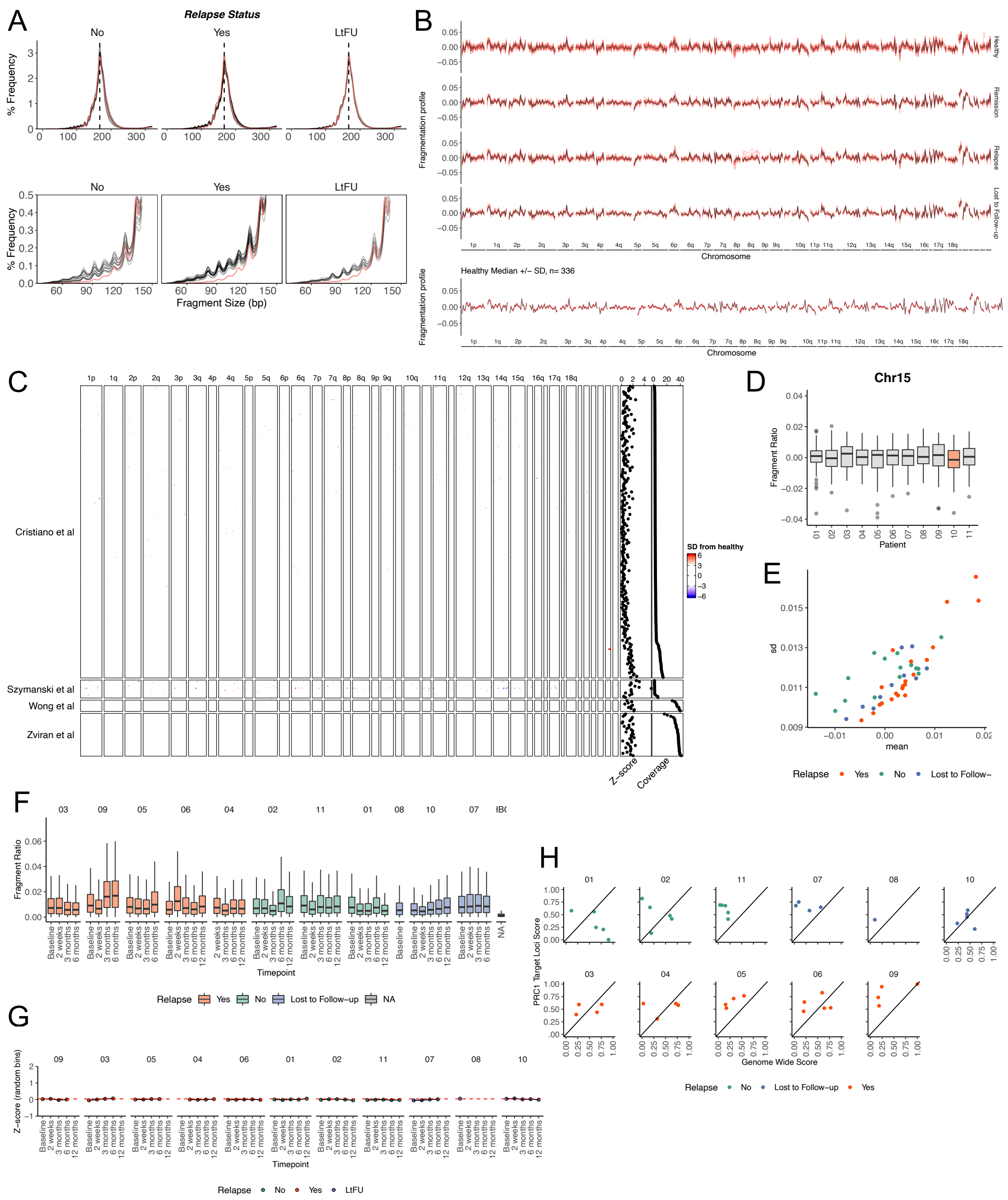

Supplemental Figure 2:

A) Fragment size frequency distributions comparing patients that did relapse versus patients that did not. The healthy median is displayed in red.

B) Fragment ratio profiles for healthy controls and our cohort separated by clinical outcome.

C) Heatmap of healthy control fragment ratio profiles showing distance from the median, genome-wide z-score, and sequencing coverage.

D) Boxplots comparing the fragment ratios along chromosome 15 in each patient. Patient 10, who has a CHIP-associated gain in chromosome 15 is highlighted in red.

E) Comparison of the mean and standard deviation of fragment ratios overlapping with PRC1 target regions.

F) Boxplots showing the fragment ratios of PRC1 target loci.

G) Z-scores calculated using fragment ratios from randomly selected 100kb bins.

H) Comparison of the genome-wide fragment ratio score (x-axis) and the PRC1 target loci fragment ratio score (y-axis).
