# Supplementary material for: Integrated, Longitudinal Analysis of Cell-free DNA in Uveal Melanoma
Source: Cancer Res Commun. 2023 Feb 15;3(2):267–80. doi: 10.1158/2767-9764.CRC-22-0456 (PMC9973415; doi:10.1158/2767-9764.CRC-22-0456)
Supplement: Figure S3 — Supplemental Figure 3 [file crc-22-0456-s09.pdf]

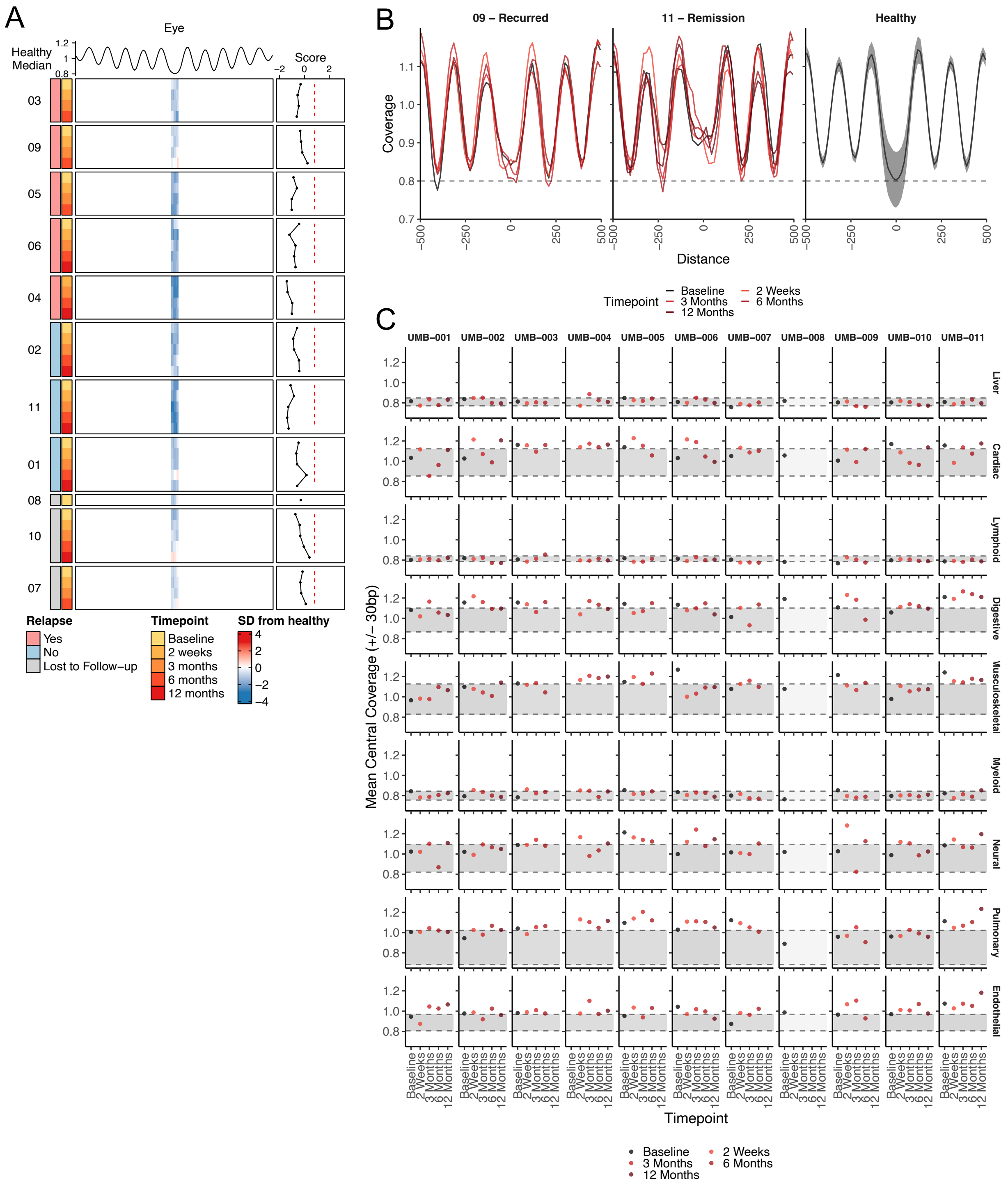

Supplemental Figure 3:

A) Nucleosome occupancy profiles associated with open chromatin sites in the embryonal eye.

B) Nucleosome occupancy tracks for one patient that relapsed, one patient that did not relapse, and a cohort of healthy controls for open chromatin sites in the embryonal eye.

C) Mean central coverage (+/- 30 bp) at open chromatin sites associated with an array of normal tissue compartments
