# Supplementary material for: Integrated, Longitudinal Analysis of Cell-free DNA in Uveal Melanoma
Source: Cancer Res Commun. 2023 Feb 15;3(2):267–80. doi: 10.1158/2767-9764.CRC-22-0456 (PMC9973415; doi:10.1158/2767-9764.CRC-22-0456)
Supplement: Figure S4 — Supplemental Figure 4 [file crc-22-0456-s10.pdf]

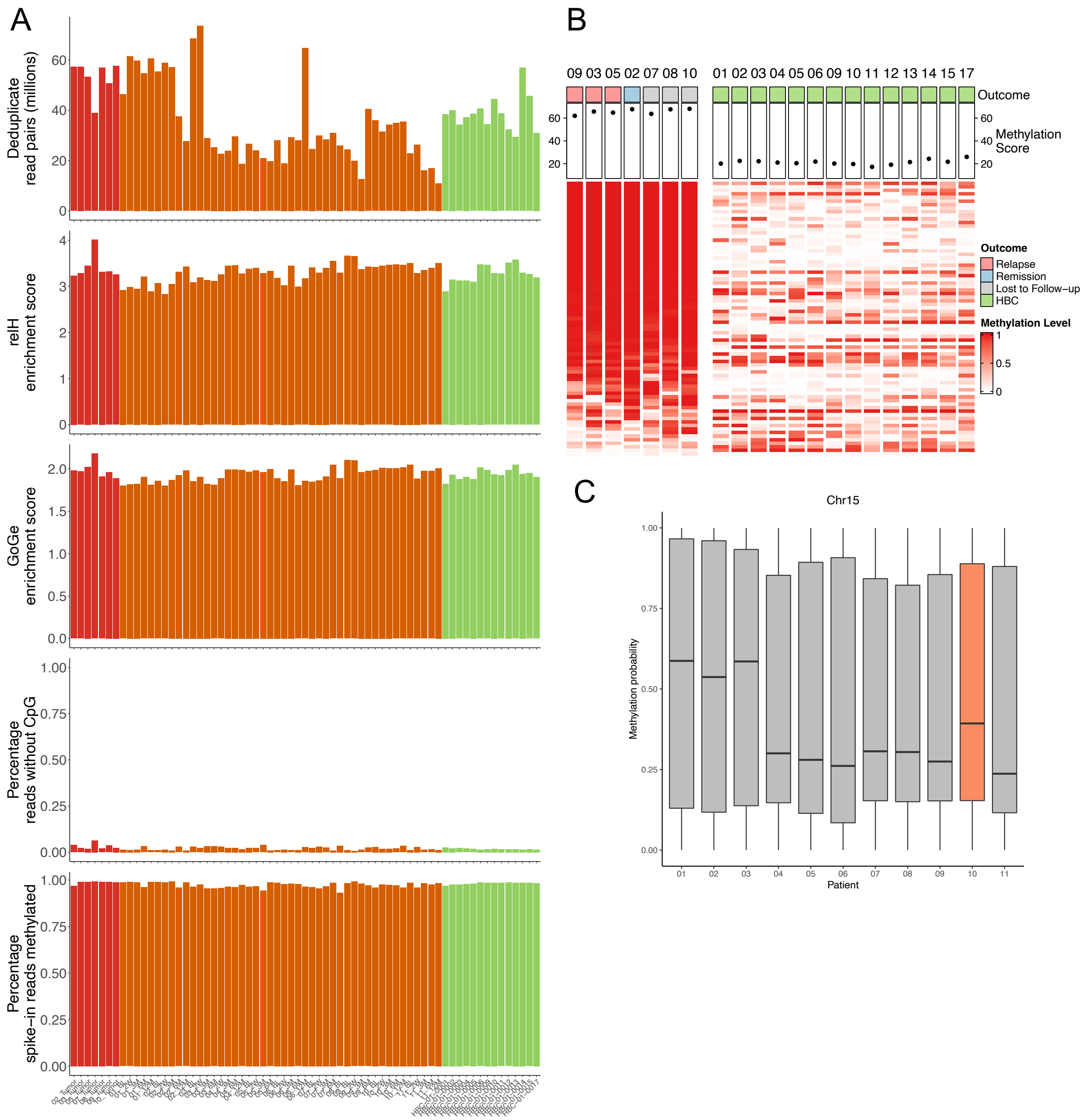

Supplemental Figure 4:

A) Quality Control metrics for cfMeDIP-seq showing # of duplicate reads in library, CpG enrichment scores (relH, GoGe), percentage of reads without a CpG, and percentage of spike-in control reads that were methylated.

B) Heatmap showing enrichment for uveal melanoma specific methylation signal in matched patient tumors and healthy blood controls.

C) Boxplots comparing the methylation probabilities from bins along chromosome 15 in each patient. Patient 10, who has a CHIP-associated gain in chromosome 15 is highlighted in red.
