# Supplementary material for: Integrated, Longitudinal Analysis of Cell-free DNA in Uveal Melanoma
Source: Cancer Res Commun. 2023 Feb 15;3(2):267–80. doi: 10.1158/2767-9764.CRC-22-0456 (PMC9973415; doi:10.1158/2767-9764.CRC-22-0456)
Supplement: Figure S5 — Supplemental Figure 5 [file crc-22-0456-s11.pdf]

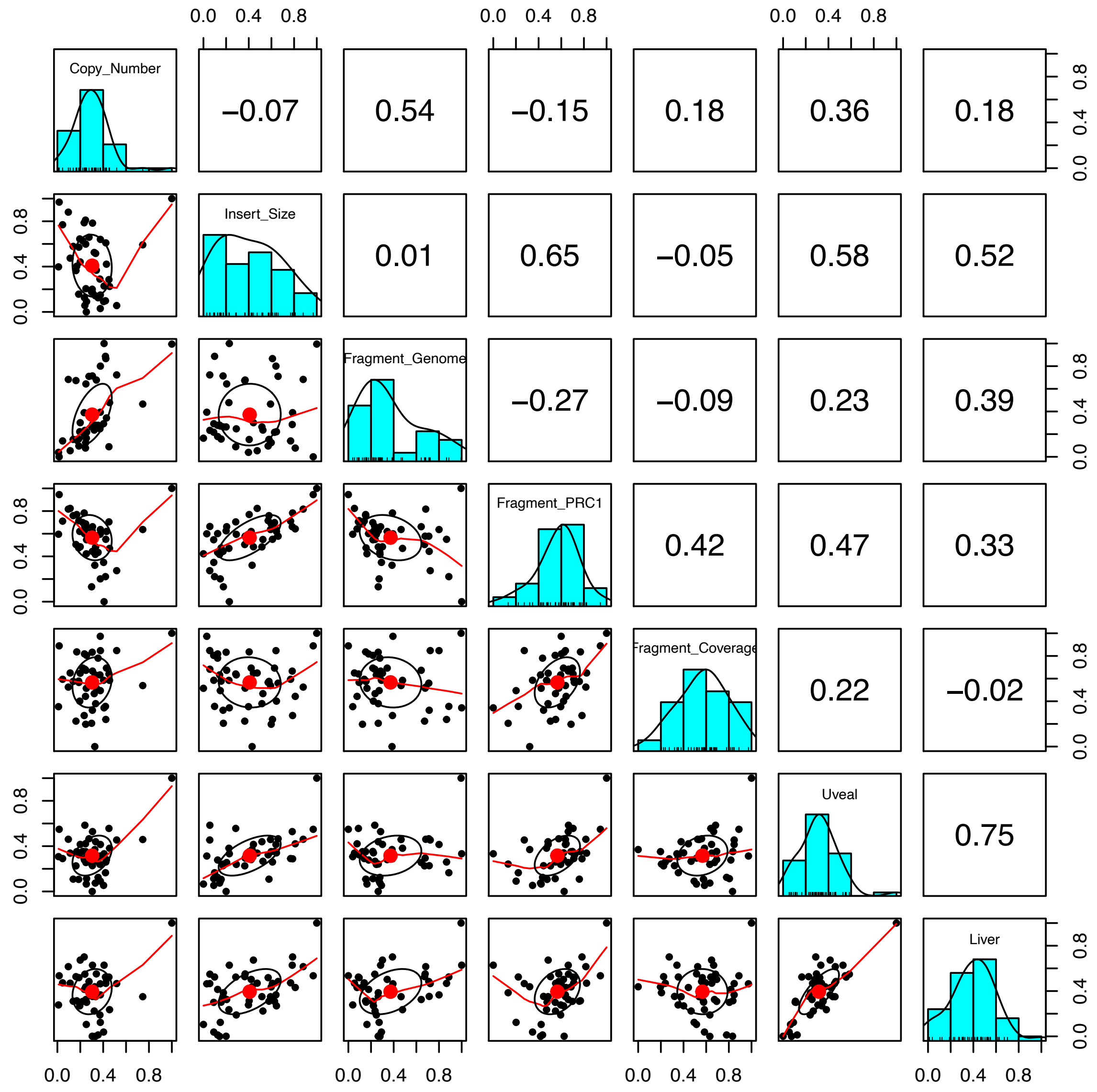

Supplemental Figure 5:
Pair-wise comparisons between scores generated from individual analyses.
